# A full-system direct weighing method with diet-stratified sampling for hospital food waste quantification

**DOI:** 10.1016/j.mex.2026.104233

**Published:** 2026-10-24

**Authors:** Tianyu Zhang-Jin, Djamel Rahmani, José M. Gil

**Affiliations:** a Universitat Politècnica de Catalunya - Barcelona Tech (UPC), Spain; b Centre de Recerca en Economia i Desenvolupament Agroalimentari UPC-IRTA (CREDA), Spain; c Doctor HC, Universidad del Bío-Bío, Chile

**Keywords:** Plate waste, Kitchen waste, Mixed methods, Healthcare sustainability, Institutional food service

## Abstract

Direct weighing enables precise quantification of hospital food waste, but its implementation across an entire hospital food service system presents substantial operational challenges. This article presents a comprehensive measurement methodology aligned with the definitions established in the Commission Delegated Decision (EU) 2019/1597 and demonstrates its application across inpatient, staff canteen, and public restaurant services in a 1,000-bed tertiary hospital in Spain. Two 7-day measurement campaigns were conducted in March 2024 and February-March 2025. The methodology integrates direct weighing of kitchen and plate waste, proportional stratified sampling by inpatient diet, food category classification, and sensitivity analyzes. It enables the estimation of edible food waste intensity per meal served, system inefficiency ratios, annualized waste quantities, and diet-specific waste generation. By detailing the sampling framework, operational procedures, data-processing methods, and quality-control requirements, this study provides a reproducible and adaptable approach to food waste quantification in complex healthcare food service systems.

• Full-system direct weighing covers kitchen and plate waste across inpatient, staff, and public food services.
• Proportional stratified sampling enables diet-specific estimates of inpatient food waste.
• The method generates comparable waste indicators by sector, service, waste origin and food category.

## Graphical abstract

**Figure:**
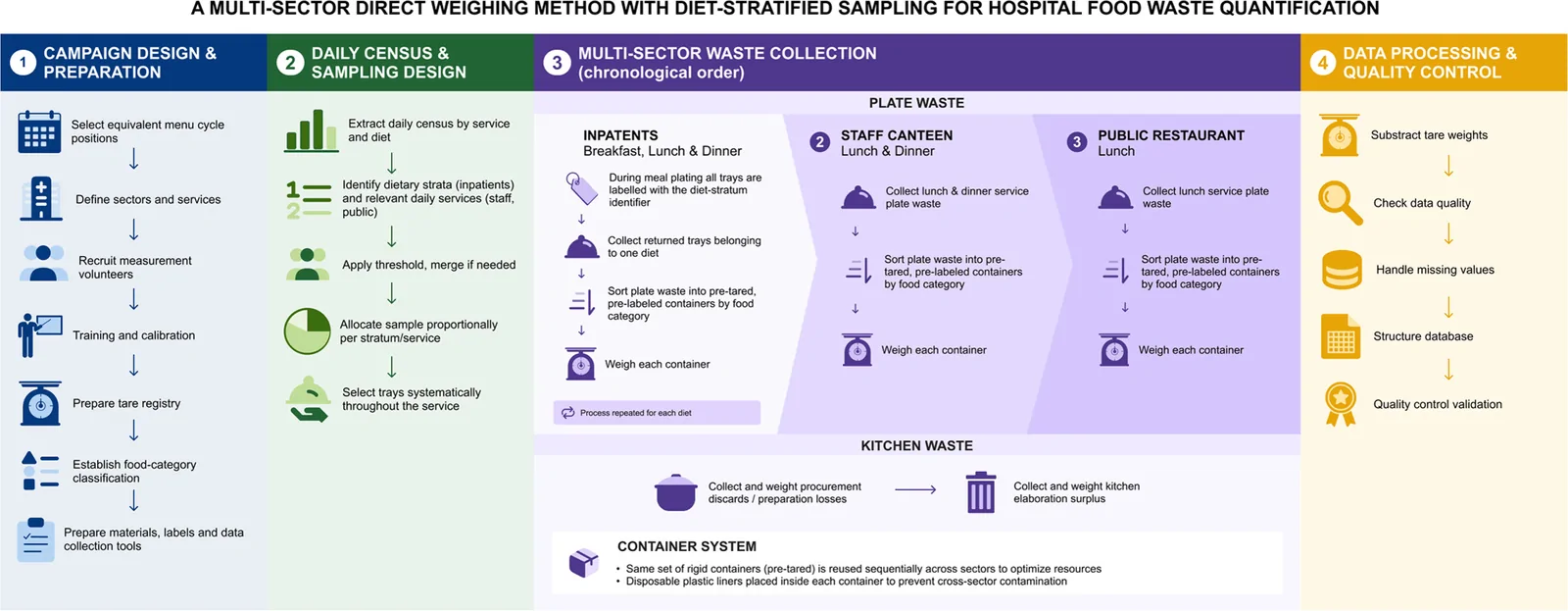

## Specifications table

**Table:** 

**Subject area**	*Environmental Science*
**More specific subject area**	Food waste
**Name of your method**	Full-system direct weighing method with diet-stratified inpatient sampling
**Name and reference of original method**	EU Commission Delegated Decision 2019/1597 of 3 May 2019 supplementing Directive 2008/98/EC of the European Parliament and of the Council (European Commission, 2019).
**Resource availability**	The data collection forms are available from the corresponding author upon request.

## Background

Hospital food waste arises both during preparation and from food left after service, and these streams need to be measured separately to account for their different underlying causes. However, few published hospital studies have measured both streams across all food-service sectors at the same time [[1], [2], [3]].

Several measurement methodologies exist for hospital food waste quantification, differing in their demands on time, cost, precision, objectivity, and reliability. Table 1 summarizes the comparative evaluation criteria defined by Xue et al. [4], which underpins the methodological choice made in this study. Direct weighing and composition analysis are the only methods rated high on all five criteria. Although both require higher time and cost investment, they are the only approaches that provide data with sufficient precision and objectivity to identify critical control points within a complex multi-sector food system.

**Table 1:** Comparative evaluation of food waste quantification methodologies [4].

Method	Time	Cost	Precision	Objectivity	Reliability
Direct weighing	High	High	High	High	High
Composition analysis	High	High	High	High	High
Surveys	Medium	Medium	Medium	Medium	Medium
Diaries	High	Medium	Medium	Medium	Medium
Photography	Low	Low	Medium	Medium	Medium
Kitchen container	Low	Medium	Medium	Medium	Medium

Existing measurement practices rely predominantly on visual estimation of plate waste, in which trained observers score tray content against a residual consumption scale. The approach is easy to operate but is affected by differences between observers and usually does not yield weights for individual food categories [5]. Gravimetric methods use calibrated scales and allow the separation of waste by category, service, and point of origin [6]. Despite these advantages, direct weighing has rarely been implemented at full-system scale because it requires substantial staff time, equipment and coordination, particularly when several hospital food services are measured together.

Inpatient sampling is an additional problem because hospitals serve standard, therapeutic, texture-modified, and pediatric diets, among others, at the same time. Treating inpatient meals as a single population prevents diet-specific waste estimates and can bias extrapolation if the sampled trays do not reflect the daily census. To address this limitation, the method introduces stratified proportional allocation by diet type. This provides diet-specific estimates without exceeding the time available during routine services.

The resulting methodology combines full-system direct weighing in three food-service sectors (inpatients, staff canteen, and public restaurant), with proportional inpatient sampling by diet. It was developed to provide hospitals and researchers with a reproducible approach for quantifying food waste across the entire food service system. The method can be used to establish baseline waste levels, locate the main sources of waste, evaluate intervention effectiveness, compare performance across hospital sectors, and support sustainability reporting and food waste reduction strategies. Although developed in a large tertiary hospital, the workflow can be adapted to other healthcare institutions operating centralized or semi-centralized food service systems.

## Method details

For this study, food waste was operationally defined as all food prepared for the intended consumers (inpatients, staff and restaurant users) but not consumed by them. This definition applied across all measured food-service nodes and included both unserved kitchen surplus and food remaining on consumers' plates.

The hospital was a 1,000-bed tertiary teaching institution in Barcelona, Spain, with two centralized kitchens. One kitchen prepared meals for inpatients, while the other, operated by an external catering company, supplied both the staff canteen and the public restaurant. Both kitchens operated a cook-serve system, in which meals were prepared and distributed for consumption shortly after cooking. The measurement methodology therefore encompassed three food service sectors across two production facilities. Food waste was quantified at designated measurement locations according the operational characteristics of each sector. Inpatient and staff plate waste were weighed at the inpatient kitchen, while staff kitchen waste was weighed at the catering contractor's kitchen inside the hospital. Both plate and kitchen waste from the public restaurant were weighed on-site. Measurement activities were coordinated according to a predefined daily schedule aligned with meal-service operations and waste collection times.

Fig. 1 shows the sequence of campaign preparation, sampling, data collection across inpatient, staff canteen and public restaurant sectors, kitchen waste quantification and data processing. Each stage is described below.

**Fig. 1:**
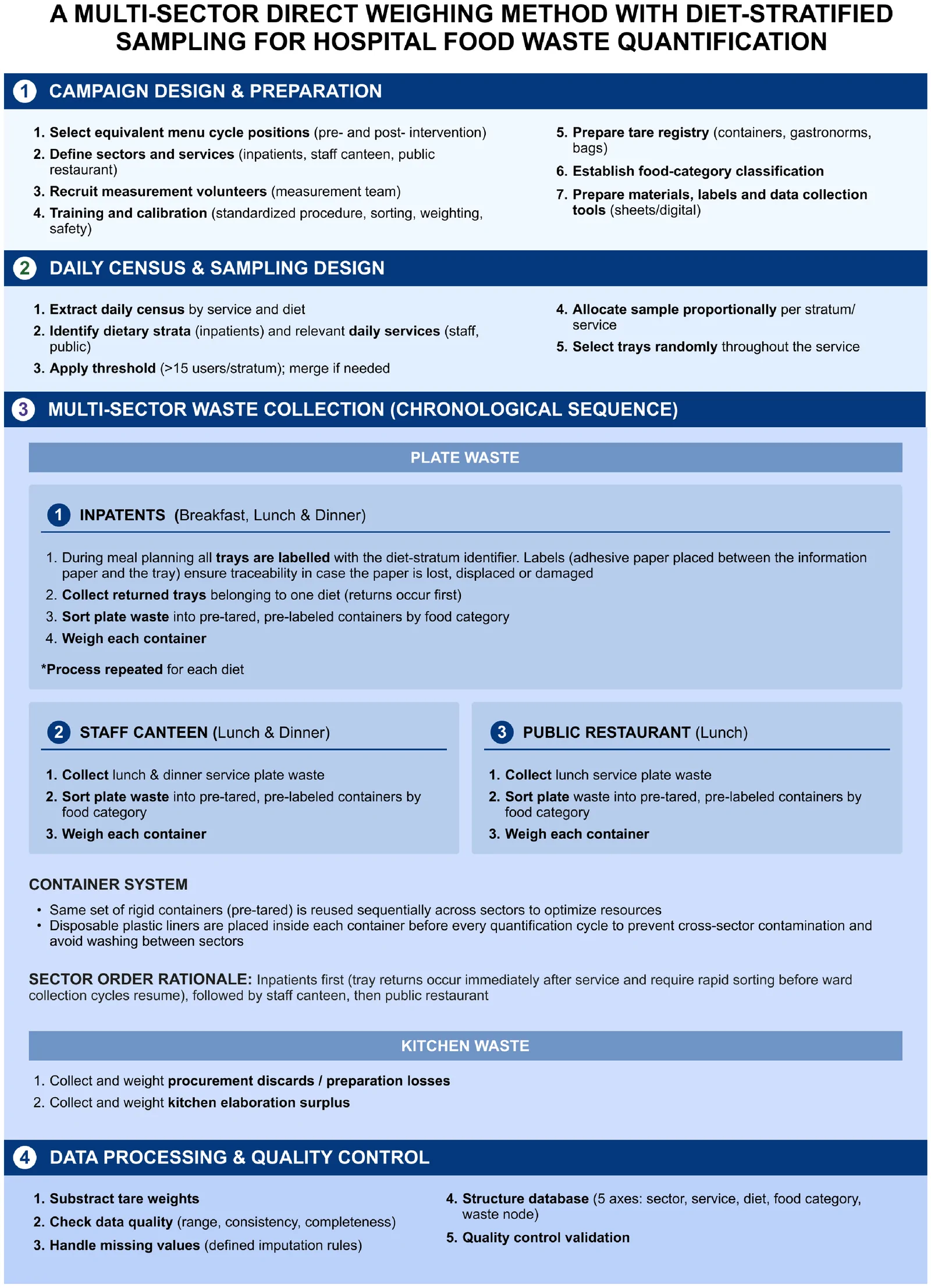
Food waste quantification workflow.

## Campaign design and timing

Two independent one-week measurement campaigns were conducted at equivalent positions in the 14-day menu cycle to control for menu-driven variation in waste composition.

• Campaign 1 (Baseline): 11–17 March 2024, covering menu cycle days 9–14 and day 1 of the following cycle.
• Campaign 2 (Post-intervention): 25 February-3 March 2025, covering the same menu cycle days 9–14 and day 1.

Scheduling both campaigns at the same point in the menu cycle reduced variation caused by different menu items. Both campaigns took place during routine operations and excluded public holidays. Between campaigns, the hospital introduced the COQUUS digital food management system in inpatient wards. Previous hospital studies have used gravimetric measurement before and after food-service interventions [7,8]; here, this design was applied across the full food system and added diet-stratified sampling in 2025.

In parallel with the COQUUS system implementation, three types of targeted awareness-raising materials were designed and deployed across the hospital prior to the 2025 campaign, each adapted to the user group (inpatient, kitchen staff, non-patient consumers).

## Equipment and materials

• **Calibrated electronic platform scales**: minimum capacity 30 kg, readability ≥1 g. Scales were tared before each measurement session and verified daily against a reference weight.
• **Pre-tared, pre-labeled collection containers**: one container per food category. Tare weights were recorded at the start of each campaign and verified mid-campaign.
• **Standardized food category classification**: pre-established classification guide with photographic references distributed to all measurement teams.
• **Pre-registered gastronorm tare weights**: recorded prior to the campaign for kitchen waste containers used in elaboration surplus weighing.
• **Patient census records:** daily census data per dietary variant downloaded from the hospital information system before each measurement day to establish the stratified sampling frame for inpatients.
• **Data collection forms**: standardized paper forms completed at the collection point with transcription to electronic database at the end of each measurement day.
• **Measurement team**: minimum 5 trained personnel per service for inpatient and staff collection; 3 per public restaurant. Team members received standardized training covering food category classification, edible/inedible separation, tare verification, weighing procedures, data recording procedures, and hospital hygiene requirements. Team size should be adapted to the sampling requirements, service volume, and time available for waste sorting and quantification.
• **Pilot exercise**: before field data collection, a pilot exercise was conducted to validate all measurement procedures, verify the functionality and availability of equipment and materials, and ensure consistency among team members in waste sorting, weighing, and data-recording practices. Any discrepancies identified during the pilot exercise were discussed and corrected before the start of data collection.

## Operational sequencing of sector quantification

Measurements were organized according to the order in which trays returned to the collection area (Fig. 1). At the inpatient kitchen, inpatient plate waste was processed following tray return from the wards, while staff plate waste was quantified according to its respective return schedule. Kitchen waste and public restaurant waste were measured at their designated locations, with activities coordinated to accommodate routine food service operations.

The same pre-tared rigid containers were reused in the three sectors. Before each collection period, the team placed a new disposable liner in every container. Once a sector had been measured, the liners containing the sorted waste were removed and replaced before trays from the next sector were processed. This procedure reduced the number of containers required, kept waste from different sectors separate and prevented cross-sector contamination. A daily measurement schedule was established in coordination with hospital kitchen managers and the catering service provider, taking into account meal preparation, tray-return times, kitchen waste collection, and personnel availability. The schedule specified the timing and sequence of quantification activities across the three food-service sectors to minimize interference with routine operations (Table 2).

**Table 2:** Daily operational schedule for tray labeling and food waste quantification across hospital food service sectors.

Service	Time	Food waste quantified	Location
Breakfast	07:45–09:00	Inpatient tray labeling	IK
	09:45	Adult inpatient plate waste	IK
	10:30	Staff kitchen waste (previous supper)	CK
Lunch	11:30–13:15	Inpatient tray labeling	IK
	13:30	Inpatient kitchen waste	IK
	13:50	Staff plate waste	IK
	14:30	Inpatient plate waste	IK
	15:30	Public restaurant plate waste	PR
	16:00	Public restaurant kitchen waste	PR
	16:20	Staff kitchen waste	CK
Supper	17:30–19:15	Inpatient tray labeling	IK
	19:30	Inpatient kitchen waste	IK
	20:00	Public restaurant kitchen waste (bakery)	PR
	20:15	Inpatient plate waste	IK
	22:00	Staff plate waste	IK

Note: IK: Inpatient kitchen: CK. Contractor’s kitchen. PR: public restaurant. The schedule reflects the operational arrangements of the study hospital. Measurement times, locations, and personnel allocation should be adapted to each institution’s meal-service organizsaztion, production system, sampling requirements, and available resources.

## Inpatient plate waste collection

During meal plating, all trays were labeled with the corresponding diet-stratum identifier to ensure post-consumption traceability during tray return. Although trays already contained a paper label with dietary and meal information, these labels could be lost, displaced, or damaged during tray distribution, consumption, or return handling. To ensure reliable identification during quantification, an additional adhesive label was placed between the information sheet and the tray structure, securing tray traceability throughout the collection process.

Inpatient plate waste was measured immediately after tray return from wards, prior to any dishwashing, to prevent partial waste removal by kitchen staff. The following procedure was repeated for each diet stratum and service:

• **Step 1.** At tray return, collect the designated number of trays per diet and service from the tray return conveyor before dishwasher entry.
• **Step 2.** For each tray, scrape the plate waste into the corresponding food-category container. The complete food category classification used for inpatient tray sorting is specified in Table 3.

**Table 3:** Food categories collected per service.

Patient — Breakfast	Patient — Lunch/Supper
Cereals	Tomato
Cheese	Soup
Jam	Legumes
Pastry / bakery	Pasta
Porridge (papilla)	Rice
Tomato	Vegetables
Sandwiches	Meat
Biscuits	Fish
Butter	Salad
Fruit	Eggs
Dairy products	Potato / tubers
Bread	Vegetable protein
Compote	Fruit
—	Dairy products
—	Bread
—	Puree
—	Compote

Note: The food categories presented reflect the menu composition and food service characteristics of the study hospital. They may be adapted to other institutional contexts while retaining sufficiently broad and consistent categories to facilitate comparisons across food service settings and studies.
• **Step 3.** Inedible fractions (bones, rinds, shells) are separated into a dedicated inedible container during scraping.
• **Step 4.** At the end of each diet waste separation, weigh each food-category container on the platform scale. Record gross weight. Subtract pre-registered tare weight to obtain net waste weight per category.
• **Step 5.** Record on the standardized form: date, service, sector, diet, food category, gross weight, tare weight, net weight, tray count.

  

**Inpatient stratified proportional sampling** (2025 advance)  

Target sample sizes were determined with hospital food service managers and staff responsible for tray returns. The pilot exercise assessed how many trays the team could sort and weigh before they entered the dishwashing process. For inpatient services, a target of 100 trays per meal could be allocated across the diet strata without delaying routine kitchen operations. Larger samples for weekday staff lunches were used because more consumers and returned trays were available. The targets were lower for staff suppers and public restaurant lunches because these services had fewer consumers. The final targets therefore reflected the volume of each service and the staff, equipment, and time available for measurement.

For inpatients, in 2024, trays per service were selected by random selection after kitchen return, without stratification by diet type. In 2025, proportional stratified sampling was introduced Table 4.

**Table 4:** Sampling design by sector and service, 2024 and 2025.

Sector / Service	2024 n (trays/day)	2025 n (trays/day)	Selection method 2024	Selection method 2025
Inpatients — Breakfast	100	100 (stratified)	Random post-return	Proportional by diet stratum
Inpatients — Lunch	100	100 (stratified)	Random post-return	Proportional by diet stratum
Inpatients — Supper	100	100 (stratified)	Random post-return	Proportional by diet stratum
Staff — Weekday lunch	150	150	Random post-return	Random post-return
Staff — Weekend lunch	70	70	Random post-return	Random post-return
Staff — Supper	50	50	Random post-return	Random post-return
Public restaurant — Lunch	15	15	Random post-return	Random post-return

***1. Daily census query***

At 07:00 on each measurement day, extract the current census per dietary variant. For each of the 10 diet strata (see Table 5), record the number of active patients. Exclude patients marked as nil-by-mouth (NPO), on parenteral nutrition, or discharged prior to breakfast service.

**Table 5:** Inpatient diet strata for 2025 stratified proportional sampling.

No.	Stratum (diet type)	Clinical description
1	Basal	Standard unrestricted diet for ambulatory or mildly ill patients
2	Emergency basal	Short-stay patients admitted via emergency with no specific dietary restriction
3	Pediatric basal	Age-adapted standard diet for pediatric inpatients
4	Carbohydrate-controlled	Diabetic or glycemic-management diet with restricted simple carbohydrates
5	Fat-controlled	Low-fat diet for hepatobiliary, cardiac, or lipid-related conditions
6	Protein-controlled	Restricted protein intake for renal insufficiency or hepatic encephalopathy
7	Easy digestion	Low-residue, low-fat, bland diet for gastrointestinal or post-surgical recovery
8	Residue/fiber-controlled	Diet restricting insoluble fiber for inflammatory bowel conditions or colonoscopy preparation
9	Texture-modified / pureed	Mechanically altered preparations for dysphagia, oro-pharyngeal surgery, or mastication impairment
10	Liquid / semi-liquid	Full-liquid or semi-liquid diet for severe dysphagia, post-operative fasting transitions, or oral surgical recovery

Note: The diet categories and their distribution reflect the dietary provision of the study hospital and may differ across institutions. When applying the methodology elsewhere, sampling strata should be adapted while preserving proportional representation of the relevant diet groups.

***2. Minimum threshold application***

Before applying the threshold, consolidate clinically similar dietary variants into the 10 analytical strata shown in Table 5. Only dietary strata with 15 or more active patients in the daily service census were included in the proportional allocation. Diet categories with fewer than 15 patients per service were excluded from the sampling frame and from the corresponding meal-count denominator. This threshold prevents allocation of zero or near-zero tray counts to low-frequency diets, which would have produced unstable diet-specific waste estimates. The exclusion rule is applied per service and per day and all exclusions are documented on the field form. In practice, during the 2025 campaign, all 10 diet strata retained for analysis met the minimum threshold on every measurement day and were sampled independently, with the basal diet stratum receiving the largest allocation of trays.  

***3.* Proportional allocation**

For each qualifying stratum *i*, calculate the target tray count:

$$n^{i}=\text{round}\left(\frac{100\;\times \;N_{i}}{\Sigma \;N_{i}}\right)$$

where *N*^i^ is the stratum census. Round to the nearest integer. After rounding, minor discrepancies may occur such as that Σ*n*^i^ ≠ 100. These discrepancies were corrected by adding or subtracting trays from the largest stratum until the total allocated sample size equalled 100 trays per service.

Example:

If the daily census is:

Basal = 440 patients; diabetic = 150 patients; pureed = 100 patients; other diets = 310 patients.

Total census = 1000 patients.

Basal allocation: n= round (100 × 440/1000) = 44 trays.

## Staff and public restaurant plate waste collection

In the staff canteen and public restaurant, trays were returned by users to a centralized return station. Plate waste sampling was performed by selecting trays at random from the return stream throughout the service period.

Target sample sizes were predefined for each sector and service: 150 trays for staff weekday lunches, 70 for staff weekend lunches, 50 for staff suppers, and 15 for public restaurant lunches.

Sampling was conducted continuously throughout the service to ensure that trays from the entire service period were represented in the sample.

Selected trays followed the same sorting, weighing, and recording procedures used for inpatient plate waste collection. Inedible fraction separation was applied identically. The food category classifications used for the staff canteen and public restaurant are presented in Table 6. The lists differ from the inpatient lists, reflecting differences in menu structure (e.g., the staff and public sectors include couscous; the public sector includes sandwiches, and pastries as separate categories absent from clinical menus).

**Table 6:** Food categories collected from the staff and public sectors.

Staff canteen	Public restaurant
Tomato	Bread
Soup	Sandwich
Legumes	Pastry
Pasta	Vegetables
Rice	Pasta
Vegetables	Meat
Meat	Potato / tubers
Fish	Puree
Salad	Fish
Eggs	Salad
Potato / tubers	Legumes
Vegetable protein	Couscous
Couscous	Eggs
—	Fruit

Note: As with the inpatient food categories, this classification may be adapted to other institutional contexts while maintaining sufficiently broad and consistently defined categories to facilitate comparisons across settings and studies.

## Kitchen waste collection

Kitchen waste was measured separately from plate waste. At the end of each service, the remaining food was weighed in its original gastronorm pans (container plus contents), without transfer to category-specific containers.

Net elaboration surplus weight was calculated as:

$$W_{net}=W_{gross}-W_{tare}$$

where:

W_net_= net kitchen waste weight;
W_gross_= weight of the gastronorm pan and its contents;
W_tare_= pre-registered tare weight of the empty gastronorm pan.

Food items were subsequently classified according to their principal ingredient using the corresponding technical menu sheets for each service and menu position. Tare weights for each gastronorm container type were pre-registered, visually coded, and verified at the start of each campaign.

## Data processing and quality control

Following each measurement day, all raw weighing records were reviewed for recording errors, internal inconsistencies (e.g., gross weight lower than tare), and anomalous values before entry into the analysis database. Implausible net weights were checked against the original paper forms and field notes.

The cleaned dataset was structured across five analytical axes to support the indicator calculations and statistical comparisons described below: (1) day of quantification; (2) sector (inpatient services, staff canteen, public restaurant); (3) meal service (breakfast, lunch, supper); (4) food category; and (5) waste origin (plate waste or kitchen waste). These variables allowed each observation to be traced from the field record to aggregate results and supported any level of disaggregation required for sub-group analysis or sensitivity testing. Data processing, structured entry, and preliminary quality checks were performed in Microsoft Excel. Final statistical analysis was performed in R.

## Derived indicators

The following indicators are calculated from the raw weight and census data:

**• Edible food waste ratio (EFW/TFW ratio, %):**

$$EFWR=\frac{EFW}{TFW}\times \;100$$

where EFWR is the edible food waste ratio; EFW is the mass of edible food waste, and TFW is the total food residue generated. Calculated per sector and for the combined system.

**• Edible waste intensity (g/meal served):**

$$I=\frac{\left(EFW\times \;1000\right)}{M}$$

where *M* is the number of meals served. Calculated per sector, service and diet stratum.

**• System inefficiency ratio (%):**

$$SI=\;\frac{EFW}{P}\;\times \;100$$

where *P* is the total food production weight, including all food discarded for service and all measured elaboration surplus. Calculated per sector and campaign.

**• Annual projection (kg/year):**

$$AP=\;\bar{EFW_{week}\times \;52.14}$$

where $\bar{EFW_{week}}$ is the total edible food waste generated during the 7-day measurement campaign. Applied to each measurement campaign separately.

## Plate waste extrapolation formulae

Because the 2024 and 2025 inpatient sampling designs differ, total plate waste was extrapolated using different calculation methods in each campaign, and a sensitivity analysis was conducted to assess the impact of this methodological difference on aggregate estimates.

**• 2024 (non-stratified):** For each food item *I*, total plate waste was estimated as

$$PW_{i}=\left(\frac{PW_{sample,\;i}}{n_{sample,\;i}}\right)\times N_{i}$$

where:

*PW_i_* = extrapolated plate waste for food item *i*;
*PW_sample,i_* = measured plate waste of food item *i* in the sampled trays;
*n_sample, i_* = number of sampled consumers receiving food item _i_,
*N_i_*= number of consumers receiving food item *i* during the service

**• 2025 (diet-stratified):** For each diet stratum *d*, total plate waste was estimated as

$$PW_{d}=\left(\frac{PW_{sample,\;d}}{n_{sample,\;d}}\right)\times N_{d}$$

where:

*PW_d_* = extrapolated plate waste for diet stratum *d*;
*PW_sample,d_* = measured plate waste in sampled trays belonging to diet stratum *d*;
*n_sample, d_* = number of sampled consumers in diet stratum *d*,
*N_d_*= number of consumers in diet stratum *d* during the service.

The stratified calculation reduces uncertainty arising from the unknown diet composition in the 2024 sample, and permits waste to be estimated for individual diet groups.

## Statistical analysis

Temporal comparisons between the two campaigns were performed at the daily observation level (*n* = 7 daily values per campaign per sector/indicator). Differences in daily edible-waste intensity (g/meal served) between the 2024 and 2025 campaigns were evaluated using the Wilcoxon two-sided rank-sum test, a non-parametric procedure appropriate for small samples and non-normal data distributions. These analyzes were used to evaluate the protocol in this hospital and are not required components of the measurement protocol in other settings.

Non-parametric bootstrap confidence intervals (95%) were computed for mean edible-waste intensity differences using *B* = 10,000 resamples and a fixed random seed of 123. Effect size was estimated using Cliff’s *delta.*

Three pre-specified sensitivity analyzes were conducted:

• **Census harmonization scenarios:** daily edible-waste intensity was recalculated under three meal-count specifications: the recorded 2024 meal counts, the 2024 meal counts reduced by approximately 25 meals per service to represent the low frequency groups excluded in 2025, and the alternative total meal variable available for 2025. These calculations assessed whether the comparison between campaigns was sensitive to the meal-count denominator.
• **Plate waste extrapolation scenarios:** for the 2024 non-stratified inpatient sample, the meal plate-waste weight per sampled tray was decreased and increased by 20%, and total plate waste was recalculated under each scenario. The 2024 kitchen-waste estimate was held constant, and each resulting total edible-waste estimate was compared with the observed 2025 total.
• **Kitchen waste imputation strategy:** missingness was assessed by year, sector, date, and meal service. Four fully missing staff kitchen-waste services in 2025 were imputed using the mean of complete observations from the same year, staff sector and meal service. One partially recorded service retained its observed value. Sensitivity analyzes treated fully missing services as zero, restricted the comparison to complete recorded days, and applied the previous daily mean-imputation method.

## Complementary mixed method modules

Interviews, a focus group, and a patient survey were also used to help interpret the waste measurements. These optional components are not part of the weighing protocol:

• **Semi-structured interviews:** the inpatient kitchen head and the catering contractor kitchen manager were interviewed before the 2024 baseline campaign about production logic, forecasting practices, and food waste management. The interviews also informed the design of the quantification protocol. Interviews were transcribed verbatim and subjected to thematic analysis.
• **Multi-stakeholder focus group:** held on 9 May 2024 after the baseline campaign, with 12 participants representing patients, physicians, nurses, nutritionists, dietitians, quality staff, and kitchen managers. Outputs were reviewed to identify recurrent explanations of food waste generation, operational constraints and potential interventions. Findings were organised around the main discussion topics and compared across sectors.
• **Patient survey:** a structured questionnaire was administered during the 2025 measurement week. It assessed (i) food satisfaction dimensions (7-point Likert scale covering eight service attributes); (ii) reasons for not finishing meals (multiple choice); and (iii) four attitudinal constructs: *personal norm* (perceived internal moral obligation to avoid wasting food), *personal attitude* (degree to which reducing food waste is perceived as positive and desirable), *social norm* (perceived social pressure or influence regarding waste behavior), and *general ecological worldview* (beliefs about ecological balance, sustainability, and limits to human growth). Initial recruitment relied on QR codes attached to inpatient trays together with a brief explanatory leaflet; however, participation rates were very low. Hospital staff therefore administered the questionnaire directly to inpatients. Despite this approach, access to patients remained operationally constrained due to clinical workload, patient availability, health status, and ward routines. A total of 85 questionnaires were administered, of which 49 valid responses were retained. Survey data from staff and public sectors samples were too small for quantitative analysis and were excluded from statistical reporting. Associations between attitudinal variables and self-reported consumption behavior were examined using bivariate Pearson correlations.

Themes from the interviews, focus group and patient survey were synthesised into a causal loop diagram (CLD), representing hypothesised interactions among structural, operational, and human factors influencing food waste generation. The CLD was an output of this synthesis and was used to interpret the quantitative findings; it was not a pre-existing coding framework.

## Method validation

### Reproducibility

Both campaigns covered days 9–14 and day 1 of the 14-day menu cycle. This alignment reduced variation due to menu composition. The edible fraction was similar in the two campaigns, which is consistent with stable application of the edible/inedible classification. Because the campaigns were conducted only once per year, these observations do not establish measurement repeatability or reproducibility.

### Resolution and discriminatory capacity

The protocol generated waste estimates by sector, service, waste origin, food category, and diet type. Edible food waste intensities differed across the three food service sectors. Within inpatient services, baseline production inefficiency ranged from 30.6% to 73.8% across services. These differences show the additional information obtained by retaining service- and sector-level records instead of reporting only a hospital-wide total.

### Waste origin discrimination

The method distinguished kitchen waste from plate waste across all sectors and campaigns. Plate waste was the largest stream across the hospital food system, while kitchen waste also made a substantial contribution in inpatient services. Keeping the two streams separate makes it possible to identify whether a change between campaigns occurred before or after food was served.

### Validation of diet-stratified sampling

Diet-stratified sampling in 2025 made it possible to estimate waste separately for each eligible diet group, which was not possible with the 2024 sample. This distinction matters because waste rates may differ among diets. An unstratified sample may yield imprecise estimates when waste rates differ among diets and some diet groups are poorly represented. In 2025, the sample of 100 trays was allocated in proportion to the daily census of each eligible diet group. Waste was extrapolated within each group, and the resulting estimates were then combined. The results showed that waste occurred across several diet groups rather than being concentrated in one group. Stratification therefore provided both diet-specific results and a population estimate that accounted for the hospital’s diet distribution.

## Sensitivity analysis

The effect of the meal-count denominator was examined under three specifications. Under the primary harmonized denominator, inpatient edible-waste intensity decreased from 300.7 to 281.6 g/meal (6.4%). Using the recorded 2024 meal counts gave a reduction of 11.3 g/meal (3.8%), while the alternative 2025 total-meal specification gave a reduction of 19.9 g/meal (6.8%). The direction of the comparison was unchanged, and the uncertainty intervals included zero under all specifications.

Under the ±20% plate waste extrapolation scenarios, the estimated reduction in total inpatient edible food waste ranged from 3.8% to 25.6%, compared with 16.1% in the base scenario.

For the staff sector, mean edible-waste intensity decreased from 163.1 to 138.7 g/meal under the primary service-level mean imputation, a reduction of 24.3 g/meal (14.9%). The estimated reductions were 31.8 g/meal when fully missing services were treated as zero, 40.5 g/meal when the analysis was restricted to complete recorded days, and 28.8 g/meal under the previous daily mean-imputation method. All scenarios showed the same direction of change, although their confidence intervals included zero.

## Limitations

• **Single institution:** The method was developed and validated at one large tertiary teaching hospital in Barcelona. Replication at community hospitals, long-term care facilities, or hospitals with different service models (e.g., room service, cook-chill, cook-freeze) is required before the sampling parameters (100 trays/service, 10 strata) can be adopted as universal defaults. Institutions with lower census may need to adjust stratum thresholds or reduce diet-strata granularity.
• **Seasonal and operational snapshot:** Each campaign covers seven consecutive days at one position in the 14-day menu cycle. Consequently, the methodology does not fully capture variation across the complete menu rotation or potential inter-seasonal differences in food service operations (e.g., summer vs winter). For comprehensive characterization of institutional food waste patterns, future research should ideally cover the full menu cycle and, where operationally and financially feasible, replicate measurements across different seasons.
• **Denominator harmonization between campaigns:** the 2025 sampling design excluded diet categories with fewer than 15 patients per service, while the 2024 campaign included meals from all inpatient diet categories. For the comparison, the equivalent low-frequency groups from the 2024 campaign were removed. Researchers comparing campaigns that use different sampling designs should define the same population for each period and test whether alternative denominator choices change the results.
• **Limited patient-level linkage and survey representativeness** (for studies incorporating the optional patient survey component): the patient survey produced 49 valid responses despite direct administration by hospital staff. This sample was sufficient to describe patterns in patient attitudes and satisfaction but was too small to examine the causes of food-waste behavior. The questionnaires could not be linked to the corresponding measured trays because of practical and ethical restrictions in the hospital. Therefore, associations between survey responses and measured plate waste could not be assessed at the patient level. Future research could examine secure and practical methods for linking questionnaires with individual trays.

## Ethics statements

The study did not involve any intervention on patients beyond their standard clinical care. All patient participants who completed the survey provided written informed consent beforehand. Qualitative interviews and focus groups were conducted with professional staff who provided verbal informed consent. All data were anonymized prior to analysis. No personal health data were linked to food waste measurements.

## Related research article

Zhang-Jin, T., Rahmani, D., Gil J.M. (under review). Understanding hospital food waste through system dynamics: evidence from a full-system direct weighing study. *Journal of Cleaner Production.*

## Declaration of generative AI and AI-assisted technologies in the manuscript preparation process

During the preparation of this work, the authors used ChatGPT (OpenAI) to improve the language and readability and to shorten and reorganize parts of the manuscript. The authors reviewed and edited output as needed and take full responsibility for the content of the published article.

## Declaration of interests

The authors declare that they have no known competing financial interests or personal relationships that could have appeared to influence the work reported in this paper.

## Data Availability

The data supporting the findings of this study are not publicly available due to institutional confidentiality restrictions.
